# Kanamycin-Mediated Conformational Dynamics of *Escherichia coli* Outer Membrane Protein TolC

**DOI:** 10.3389/fmolb.2021.636286

**Published:** 2021-04-15

**Authors:** Biraja S. Pattanayak, Budheswar Dehury, Mamali Priyadarshinee, Suman Jha, Tushar K. Beuria, Dhananjay Soren, Bairagi C. Mallick

**Affiliations:** ^1^ Department of Zoology, Ravenshaw University, Cuttack, India; ^2^ Department of Biochemistry, University of Cambridge, Cambridge, United Kingdom; ^3^ School of Life Sciences, Ravenshaw University, Cuttack, India; ^4^ Department of Life Sciences, National Institute of Technology, Rourkela, India; ^5^ Institute of Life Sciences, Bhubaneswar, India; ^6^ Department of Chemistry, Ravenshaw University, Cuttack, India

**Keywords:** *Escherichia coli*, efflux proteins, TolC, kanamycin, molecular docking, molecular dynamic simulation

## Abstract

TolC is a member of the outer membrane efflux proteins (OEPs) family and acts as an exit duct to export proteins, antibiotics, and substrate molecules across the *Escherichia coli* cell membrane. Export of these molecules is evidenced to be brought about through the reversible interactions and binding of substrate-specific drug molecules or antibiotics with TolC and by being open for transport, which afterward leads to cross-resistance. Hence, the binding of kanamycin with TolC was monitored through molecular docking (MD), the structural fluctuations and conformational changes to the atomic level. The results were further supported from the steady-state fluorescence binding and isothermal titration calorimetry (ITC) studies. Binding of kanamycin with TolC resulted in a concentration dependent fluorescence intensity quenching with 7 nm blue shift. ITC binding data maintains a single binding site endothermic energetic curve with binding parameters indicating an entropy driven binding process. The confirmational changes resulting from this binding were monitored by a circular dichroism (CD) study, and the results showed insignificant changes in the α-helix and β-sheets secondary structure contents, but the tertiary structure shows inclusive changes in the presence of kanamycin. The experimental data substaintially correlates the RMSD, *R*_g,_ and RMSF results. The resulting conformational changes of the TolC-kanamycin complexation was stabilized through H-bonding and other interactions.

## Introduction

The Gram-negative bacteria *Escherichia coli* (*E.coli*) is a well-known human pathogen that causes urinary tract infections (UTIs), bacteremia, neonatal meningitis, and serious food-borne infections worldwide (Money et al., 2010; Pennington, 2014). The treatment of *E. coli* infection depends on its early diagnosis and scheduled administration of antibiotics. The extensive, prolonged, and unethical use of antibiotics has meant antibacterial resistance has been developed in *E. coli* strains (Cagnacci et al., 2008; Michael et al., 2014). The reasons for the emergence of antibiotic resistance has been evidenced to be associated with the overexpression of efflux pumps (Wang et al., 2001). These efflux pumps are classified into five different families: the ATP-binding cassette (ABC) superfamily, the major facilitator superfamily (MFS), the multidrug and toxic compound extrusion (MATE) family, the small multidrug resistance (SMR) family and the resistance nodulation division (RND) family (Poole, 2007; Li and Nikaido, 2009; Delmar et al., 2014). The RND family proteins, functioning as a tripartite system in *E. coli*, are composed of an inner membrane protein (IMP), a periplasmic adaptor membrane fused protein (MFP) family, and an outer membrane factor (OMF) family protein (Nikaido and Zgurskaya, 2001). The outer membrane protein (OMP) TolC works with a combination of other RND, ABC, and MFS efflux pumps to excrude drugs molecues across the lipid bilayer. Any irregularities or the absence of any of these components makes the triparty system non-functional.

In *E. coli*, the outer membrane protein TolC has long been known as a transporter of diverse small molecules and antibacterial drugs to large protein toxins (Morona and Reeves, 1981; Morona and Reeves, 1982; Morona et al., 1983). The TolC protein belongs to the outer membrane efflux proteins (OEP) family or outer membrane factor (OMF) family. The members of this family functions in conjunction with three-types of transport systems; ATP-binding cassette (ABC-type), resistance nodulation division (RND-type), and major facilitator super family (MFS-type) (Paulsen et al., 1997). The association between transporters and outer membrane efflux protein is mediated by periplasmic proteins, named membrane fusion proteins (MFPs) (Dinh et al., 1994; Zgurskaya, 2009). The inner membrane transporter AcrB and the periplasmic membrane fused protein AcrA together forms the translocase complex as AcrAB and recognizes specific substrates for efflux (Tikhonova et al., 2011) by recruiting TolC. TolC spans in the outer membrane and projects deep into the cell periplasm to export substrates out of the cell with the help of membrane-fused translocase complex and AcrAB proteins that forms the AcrAB-TolC efflux system (Fralick et al., 1996; Zgurskaya and Nikaido, 1999; Du et al., 2015). The AcrAB-TolC complex is a ΔμH^+^-dependent pump responsible for resistance to many compounds (Paulsen et al., 1996). Studies have shown that the interaction of these proteins to form the multi-protein efflux channel to export antibiotics is mediated through the conformational dynamic (Zhao et al., 2017). The recent crystal structure of TolC has confirmed the trimeric assembly of polypeptide chains with an entrance just like a cannon and a long axis of 140 Å (Koronakis et al., 2000). Each monomer is composed of 471 amino acids and 12-beta sheets (four from each monomer) which together constitute the portion of the tunnel, which is located in the bacterial outer membrane. Similarly, 12-alpha helices forming the body part of the tunnel reside in the bacterial periplasmic space and a mixed α/β-barrel as an equatorial domain. The opening and closing of the coiled-coil domain through the interactions with MFPs and IMPs initiates the efflux process. TolC is recruited to form the efflux system when the translocate complex is bound to export substrates (Sulavik et al., 2001; Koronakis et al., 2004; Li and Nikaido, 2009). The AcrAB-TolC triparty system creates a direct efflux pathway from the cytoplasm to the extracellular space and allows the efflux of substrate molecules across the cell membrane. This efflux pump has been found to contribute to the inherent resistance for ß-lactams, fluoroquinolones, aminoglycosides (Nidaido, 1994; Wang et al., 2001; Nishino et al., 2003), and other toxic compounds such as antiseptics, detergents, and dyes (Sulavik et al., 2001). Overexpression of these efflux-pump proteins have resulted in the development of antibiotic resistance bacteria (Nikaido, 1998; Li and Nikaido, 2009).

Aminoglycosides are broad-spectrum bactericide antibiotics used for the treatment of short-term infections caused by infectious bacteria such as *E. coli, Proteus* spp., *Enterobactor areogenes, Klabsiella pneumoniae, Serratia marcescens,* and *Acinetobacter* spp (Ristuccia and Cunha, 1985; Aggen et al., 2010; Landman et al., 2010). Kanamycin is a promising aminoglycoside antibiotic that has been extensively used in the treatment of *E. coli* infections worldwide. It acts on bacteria by inhibiting protein synthesis, which is essential for its survival (Zhang et al., 2015). The extensive use of kanamycin for *E. coli* treatment has developed its resistance to a greater extent and the same is considered to be brought about through the involvement of outer membrane efflux pump proteins. However, the exact mechanism of efflux and the involvement of these proteins still remains largely unknown (CarolBaker et al., 1974). It is also known that TolC-dependent efflux of antibiotics and other molecules involves the interaction of TolC with the translocase/efflux pump of two inner membrane proteins, AcrAB (Aono et al., 1998; Fralick,1996). Despite these encouraging results, the binding of antibiotics to the triparty system and their active efflux remains incomplete and needs comprehensive analysis. Hence, we aimed to explore the binding of kanamycin with TolC by molecular docking and biophysical methods, such as fluorescence spectroscopy, isothermal titration calorimetry (ITC), and conformational stability by circular dichroism (CD) studies. The conformational changes resulting from TolC-kanamycin interaction binding was established through the molecular dynamic simulation study.

## Materials and Methods

### Materials

Oligonucleotides were purchased from IDT, Singapore and the DNA polymerase, dNTPs (dATP, dGTP, dTTP, dCTP) Phusion buffer, restriction endonuclease (Sac I and Hind III), and T4 DNA ligase were purchased from NEB, USA. *Pfu* polymerase was purchased from Promega, USA. l-arabinose, protease inhibitor, lysozyme, Octyl β-D-glucopyranoside, nitrocellulose membrane, and primary antibody (anti-mouse monoclonal antibody) were purchased from Sigma-Aldrich, USA. Ni-NTA resin was procured from Qiagen. Ampicillin and kanamycin were purchased from HiMedia. Tris and phosphate buffer were purchased from MP Biomedical, LLC (France).

### Expression and Purification of TolC

TolC was effectively overexpressed in *E. coli* BL21 (DE3) competent cells and purified using a Ni-NTA affinity column. The purified recombinant TolC protein was re-purified using FPLC (Akta, Ge-Healthcare) to remove impurities (Supplementary Figure S1). BL21 (DE3) cells harboring the plasmid pBAD-*tolC* construct were allowed to grow in an orbital shaker, and the protein expression was induced with 0.2% (w/v) of l-arabinose at 37 °C for 4 h. The cell pellet obtained was resuspended in lysis buffer (20 mM sodium phosphate buffer, 150 mM NaCl, 10 µl protease inhibitor solution and 1 mg/ml Lysozyme, pH 7.5) and ruptured by passing in a French press. The unruptured cells were separated through centrifugation at 9,000 g for 30 min at 4°C, and then the membrane fraction was separated and collected at 120,000 g by using an ultracentrifuge. The pellet obtained was again resuspended in 20 ml of binding buffer (5% Triton-X-100 in Lysis buffer, pH 7.5). The solution with membrane fraction was sonicated for 20 min on ice and then centrifuged at 20,000 g for an hour at 4°C. The supernatant was mixed with Ni-NTA resin and incubated for 1 h at 4°C. Before the incubation, the Ni-NTA resin was equilibrated with 10 column volumes of binding buffer containing 2% Triton-X-100. Then the column was thoroughly washed with 10 column volume of washing buffer (20 mM sodium phosphate buffer pH 7.5, 150 mM NaCl, 20 mM Imidazole and 2% Triton-X-100). The elution was completed with elution buffer (20 mM sodium phosphate buffer pH 7.5, 150 mM NaCl, 300 mM imidazole and 2% of Triton-X-100) at a flow rate of 1 ml/min. Finally, the elute was passed through a Superdex-200 gel-filtration column pre-equilibrated with the running buffer (20 mM sodium phosphate buffer at pH 7.5, 150 mM NaCl with 1% Octyl β-D-glucopyranoside). The fractions collected were concentrated using Amicon ultrafiltration membrane and quantified using UV-visible spectrophotometer. The molecular size of the TolC protein was confirmed through the MALDI mass spectroscopy analysis (Supplementary Table S1).

### Fluorescence Study

Fluorescence quenching experiments of TolC with kanamycin complexation was carried out using a Carry Eclipse spectrofluorometer (Agilent Technologies) equipped with an external Peltier temperature controller (EC-50) to maintain the cell temperature at 25 ± 0.1°C. Prior to the stock solution preparation, 20 mM Tris-HCl buffer pH 7.2 and 1% Octyl β-D-glucopyranoside detergent were filter-sterilized and degassed to avoid scattering and the interference of dissolved oxygen. The emission spectra of TolC and kanamycin binding experiments were recorded with successive additions of increasing concentrations of kanamycin from 5 mM to 50 mM, and the spectra were recorded between 300–450 nm by keeping the excitation wavelength fixed at 280 nm. Each protein and kanamycin scan was subtracted with corresponding kanamycin concentration in buffer to avoid the dilution effect. A constant protein concentration of 5 μM was maintained throughout all the experiments to avoid data irregularities. The binding experiments were carried out with the same excitation and emission slit widths (5 nm) in an auto response time mode. Each scan was an average of five accumulations with least smoothening.

### Isothermal Titration Calorimeter Measurements

Binding of kanamycin with TolC was measured by using MicroCal PEAQ-ITC (Malvern Panalytical, United States) equipped with a temperature-controlled cell of volume 200 μl and a 40 μl micro syringe. The stock protein sample was prepared by dialyzing against 20 mM Tris-HCl buffer pH 7.2 added with 1% Octyl β-D-glucopyranoside detergent and stored at 4°C for further use. Before experiments, each solution was thoroughly degassed under vacuum (140 mbar for 10 min) to remove the dissolved air bubbles. The sample cell was loaded with 1 ml of 20 μM TolC solution and the calorimetry syringe with 1.2 mM of kanamycin in the same buffer to avoid baseline error. Both the sample and syringe solutions were added with 1% Octyl β-D-glucopyranoside detergent to avoid the dilution effects and prevent signal instability during measurements. The temperature of the sample cell and syringe for each experiment was isothermally maintained at 25°C and experiments were programmed for 20 injections with 2 μL per injection each at 300 s interval to obtain a saturated binding curve. The heat of dilution experiments of kanamycin and buffer added with 1% Octyl β-D-glucopyranoside detergent were performed in the same conditions and subtracted from the protein binding titration data (Supplementary Figure S2). Each binding isotherm was performed in triplicate and the data obtained was analyzed with MicroCal PEAQ-ITC analysis software using a single-site-binding model to determine the thermodynamic parameters viz., stoichiometry of binding (N), binding constant (*K*_*a*_), enthalpy change (Δ *H* ^o^), and entropy change (*T* Δ *S* ^o^) at temperature *T*. The change in Gibb’s free energy (Δ *G* ^o^) was calculated using the Gibb’s-Helmholtz equation:

$$\Delta G^{o}=\;\Delta H^{o}\;-\;T\Delta S^{o}$$

### Circular Dichroism Measurements

The far UV-CD spectra of TolC and kanamycin binding interaction were recorded in a Jasco-815 spectropolarimer (Jasco, Japan) using a quartz cell of 0.1 cm path length at 298.15 K. All the CD scans were performed in 20 mM Tris-HCl buffer at pH 7.2, added with 1% Octyl β-D-glucopyranoside. The blank scans of kanamycin in buffer without protein were subtracted from the protein and kanamycin in buffer scans to obtain the final spectra. For all conditions, TolC concentration was kept constant at 10 μM and the kanamycin concentration was varied from 10 to 30 μM. CD spectra were collected with a scanned speed of 50 nm/min with a fixed bandwidth of 1 nm in the range of 190–250 nm. Nitrogen gas with 99% purity was purged through the sample compartment to create an oxygen free environment. The results obtained were expressed as the mean molar ellipticity (θ):

$$\left[\theta \right]\;=\;100\;\times \;\left[\theta_{\text{obsd}}/lc\;\right]$$

Where *θ*_obsd_ is the observed ellipticity in degree, *c* is the concentration of the residues in mol/cm^3^, and *l* is the path length of the cell (Johnson, 1990).

### Molecular Docking

The sequence information and atomic coordinates of TolC protein in SDF format was obtained from Protein Data Base (PDB) of NCBI and Protein Data Bank (PDB ID:1EK9). The structural information of kanamycin was collected from PubChem database (NCBI) and Drug Bank in SDF format. Both the SDF format of TolC and kanamycin were converted to PDB format for docking purposes. Flexible docking of both the molecules were carried out through Schrodinger software (Friesner et al., 2004; Dinesh et al., 2010; Ahmad et al., 2011), and the different parameters, like bond orders, the addition of hydrogen, proper ionization state of residues, capping and termini, and so forth, were taken into consideration for the preparation of receptors. Then the refining of receptors with the H-bond assignment (water orientation, at neutral pH) and minimization of energy with OPLS 2005 force field was performed. Generation of the grid for the protein was conducted using the site surrounding the selected residues' centroids. The OPLS 2005 force filed parameters, ionization at pH 7.0 ± 2.0, stereoisomers, and generated tautomer were utilized to prepare the ligands in LigPrep (Abdullah et al., 2016). The flexible nitrogen inversion and ring conformation ligands were then docked by the Extra Precision [EP] method.

### Molecular Dynamic Simulation

All simulations were performed using GROMACS v2018.4 with the CHARMM36m force field in TIP3 water model. There was a total of 238 POPC for the top (head) and bottom (tail) bilayer with a water thickness of 17.5 Å (36,969 water molecules) from the top and bottom of the lipid head group. The systems were neutralized by adding counter ions to each of the systems. These were 115 positive sodium and 101 negative chloride ions at a 0.15 M concentration. Then the system underwent 50,000 steps of steepest descent energy minimization to remove close van der Waals force contacts. Afterward, the system was subjected to a two-step equilibration phase, namely NVT (constant number of particles, Volume, and Temperature) for 1,000 ps to stabilize the temperature of the system and a short position restraint NPT (constant number of particles, Pressure, and Temperature) for 1,000 ps to stabilize the pressure of the system by relaxing the system and keeping the protein restrained. The V-rescale temperature-coupling method was used for the NVT ensemble, with constant coupling of 0.1 ps at 303.15 K under a random sampling seed. The temperature was maintained at 303.15 K using a Nosé-Hoover thermostat with a coupling time constant of 1.0 ps. For NPT, Parrinello-Rahman pressure coupling was turned on with constant coupling of 0.1 ps at 303.15 K under conditions of position restraints (all H-bonds). Electrostatic forces were calculated for both NVT and NPT using Particle Mesh Ewald method. After equilibration, the simulation was carried out for 50 ns under the NPT ensemble without any position restraints.

All trajectory analyses were performed using the analysis tools in GROMACS package. Intrinsic dynamics stability parameters, including root-mean-square deviation (RMSD), solvent accessible surface area (SASA), radius of gyration (Rg), and root-mean-square fluctuation (RMSF) calculations, were computed using GROMACS analysis tools. Principal component analysis (PCA) or essential dynamics (ED) were performed to understand the global motion of TolC in the presence of Kanamycin in a lipid bilayer considering the main-chain atoms. Using the MD trajectory, the translational and rotational movements were removed from the complex system using *gmx cover* toolkit to construct a covariance matrix. Then, eigenvectors and eigenvalues were calculated by diagonalizing the covariance matrix. The eigenvectors that correspond to the largest eigenvalues are called “principal components (PCs)” or “eigenvectors (EVs),” as they represent the largest-amplitude collective motions. In this study we considered the first two PCs for further exploration as they represent ∼89.3% of the total motion of the complex. Clustering analysis was performed using the GROMACS tool *gmx cluster* to identify different conformational states of the complex. Further, structural superposition of the docked conformation and the representative of top ranked cluster were performed to see the variations in the ligand binding.

## Results

### Screening of Antibiotics

Antibiotics like kanamycin, tetracycline, erythromycin, chloramphenicol, norfloxacin, and rifampicin were screened for their feasible interaction with TolC using a molecular docking method to correlate their involvement in the efflux process that causes *E. coli* resistance. Molecular docking has provided valuable information and has helped to evaluate the binding patterns of six antibiotics and calculate their binding affinities toward residues in the active site of TolC. The comparative docking score and binding energies of six antibiotics estimated from the docking results of TolC are listed in Table 1.

**TABLE 1:** List of six antibiotics and their Docking scores, XP G score, and Glide G score (kcal/mol) with TolC.

Sl. No	Antibiotics	Docking score	Xp G score	Glide G score
1	Kanamycin	−6.32	−6.53	−6.53
2	Tetracycline	−5.05	−5.44	−5.44
3	Erythromycin	−4.64	−4.65	−4.65
4	Chloramphenicol	−2.88	−2.88	−2.88
5	Norfloxacin	−2.70	−2.83	−2.83
6	Rifampicin	5.92	3.63	3.63

It clearly indicates that among all chosen antibiotics, kanamycin showed a comparative strong binding affinity toward TolC with an estimated high value of free energy change, i.e., ΔG = −6.5 kcal/mol. Based on the screening results, kanamycin was chosen as the antibiotics of interest to understand its interaction with TolC and correlate the process with the efflux mechanism that causes *E. coli* resistance.

### Purification of Recombinant TolC

**FIGURE 1:**
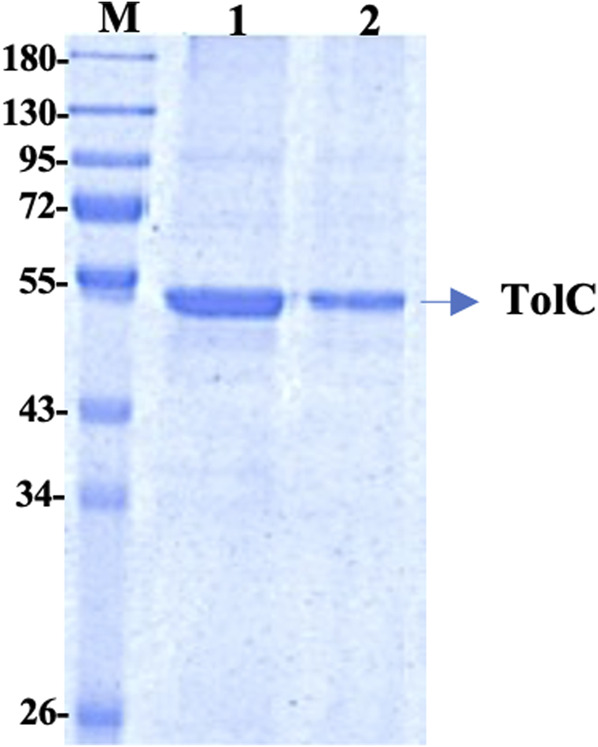
12% SDS PAGE of the purified TolC protein showing the molecular mass of ∼54 kDa. First Lane; Marker, Lane-1 and Lane-2 are purified recombinant TolC protein.

The recombinant his-tagged TolC was purified using a modified method, described previously (Koronakis et al., 1997; Koronakis et al., 2000). Purified protein concentration was determined using the DC protein assay (Bio-Rad) with bovine serum albumin as standard and the purity was checked by 12% SDS-PAGE (Figure 1), which showed a band at ∼ 54 kDa. The protein was then further purified using a FPLC, Akta, GE Healthcare to eliminate impurities. The accurate protein molecular mass was determined from the MALDI mass spectroscopy (Supplementary Table S1).

### Fluorescence Measurements

**FIGURE 2:**
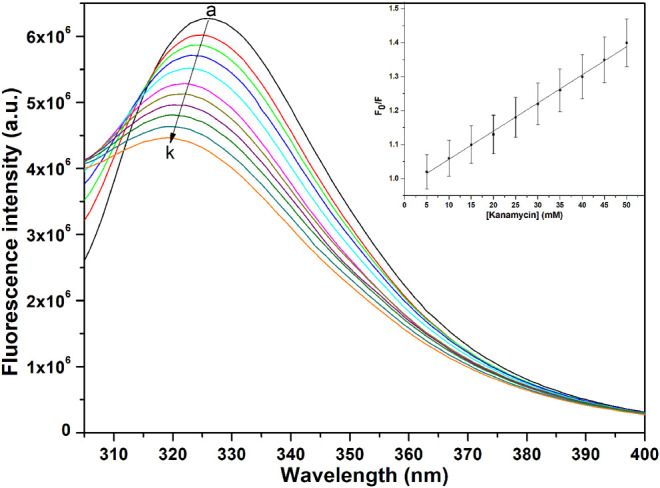
Fluorescence spectra of TolC in the presence of increasing concentrations of kanamycin monitored at 25°C. Protein in buffer as control **(A)**, and with increasing kanamycin concentrations: 5 mM **(B)**; 10 mM **(C)**; 15 mM **(D)**; 20 mM **(E)**; 25 mM **(F)**; 30 mM **(G)**; 35 mM **(H)**; 40 mM **(I)**; 45 mM **(J)**; and 50 mM **(K)** respectively. Protein concentration was kept constant at 5 µM and the experiments were performed in triplicate to obtain the best spectra. The samples were excited at 280 nm and the emission spectra were recorded in the range of 300–400 nm.

The binding affinity parameters of kanamycin with TolC was calculated using fluorescence measurements. Molecular interactions, such as formation of excited state charge-transfer complex, intersystem crossing, molecular rearrangement, and ground-state complexation, between the fluorophore and quencher can lead to fluorescence quenching (Lakowicz, 2006). TolC contains 1-tryptophan (Trp), 21-tyrosine (Try) and 10-phenylalanine (Phe) residues. All these residues can act as fluorophore to exploit the conformational changes in the neighbourhood environment of ligand binding sites in the TolC through fluorescence quenching experiments (Rabbani et al., 2011; Rabbani et al., 2013). However, as the fluorescence of tryptophan residue dominates over all others, we exclusively excite it at 295 nm to monitor its quenching effects on kanamycin binding (Ahmad et al., 2012). In Figure 2, the fluorescence emission spectra of TolC shows some change in shape with increasing kanamycin concentrations. Binding titration curves of kanamycin with TolC indicates significant decreases in the tryptophan fluorescence intensities with a blue shift of 7 nm. The inset shows the linear dependence of kanamycin on the quenching of TolC fluorescence intensities. The resulted spectra were obtained by subtracting the corresponding blanks, and the data obtained was analyzed using Stern-Volmer equation by plotting the intrinsic fluorescence intensities at λ_max_ against the [kanamycin].

$$\text{Stern}-\text{Volmer equation}:\frac{F_{o}}{F}=1+\;K_{\text{sv }}\left[Q\right]$$

Here, *F*_o_ and *F* are the fluorescence intensities in the absence and presence of kanamycin. *K*_sv_ is the Stern-Volmer quenching constant and [Q] represents the molar concentration of quencher [kanamycin]. At 298.15 K, the value of *K*_sv_ = 6.07 ± 0.05 × 10^4^ M^−1^ with *R*-value of 0.989 justifies an effective quenching process of TolC protein fluorescence intensities by kanamycin binding.

### ITC Measurements

**FIGURE 3:**
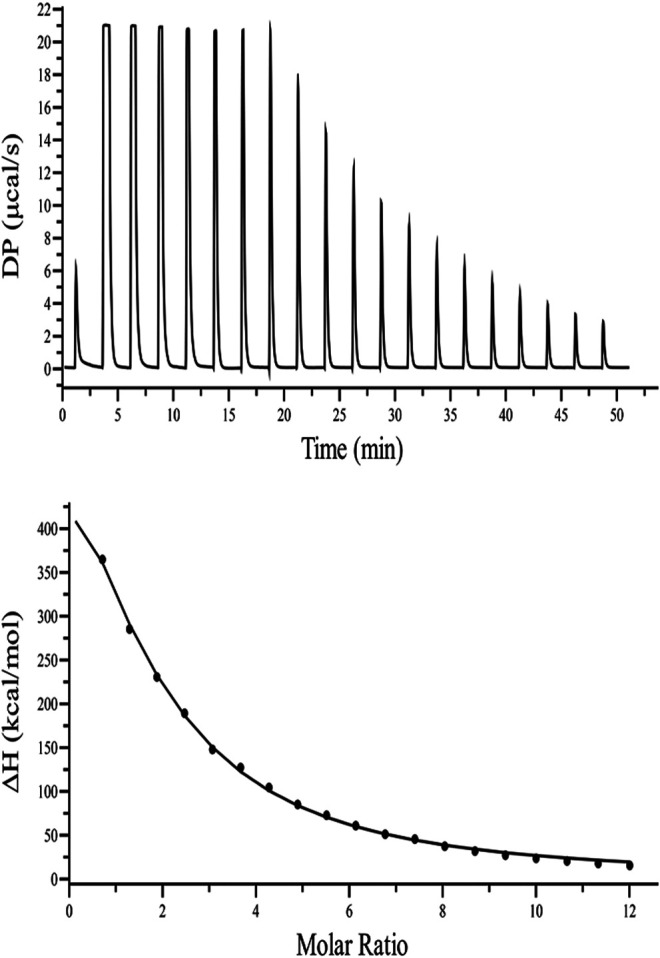
ITC binding isotherm of TolC with kanamycin at 25°C. The upper panel indicates the raw data points for heat produced with time for each titration of 1.2 mM of kanamycin with 20 µM TolC at 25°C. The lower panel shows the binding isotherm obtained after subtracting the heat of dilution with integration of peak areas and normalization to produce a plot of molecular heat change against the kanamycin/TolC molar ratio. The fitting line in the curve is shown using a black solid line.

Fluorescence quenching studies indicate that kanamycin has a high binding affinity toward TolC. And to support this binding interaction result, we performed ITC binding titration of TolC with kanamycin and calculated the thermodynamic parameters as binding constant (*K*_*a*_), enthalpy change (Δ*H*), entropy change (Δ*S*), binding stoichiometry (N), and free energy change (Δ*G*). Figure 3 shows the ITC isotherm produced from the titration of kanamycin with TolC at 298.15 K. The upper panel of binding isotherm with positive heat change indicates that an endothermic thermogram produced unfavourable binding standard enthalpy of 80.0 ± 0.9 kcal/mol and calculated favourable value of binding entropy change at *T* (K) is −88.3 ± 0.8 kcal/mol. The lower panel shows the amount of heat released with each successive injection as a function of molar ratio of TolC and kanamycin. The heat change on dilution of kanamycin into Tris-buffer was measured in the same conditions and subtracted from the heat changes by the titration of kanamycin with TolC. The overall heat changes due to the interaction of kanamycin with TolC was plotted against the molar ratio of TolC and kanamycin. The raw data obtained for kanamycin and TolC interaction was fitted to the binding models using the inbuilt origin software. The negative values of Gibb’s free energy indicates the spontaneous formation of the TolC-kanamycin complex (Rahman et al., 2019). The best fitted ITC isotherm shows a single binding site model and the thermodynamic parameters obtained are listed below in Table 2.

**TABLE 2:** Thermodynamic parameters obtained from the ITC binding of kanamycin with TolC measured at 298.15 K. The values are means of triplicates ±SD.

N	*K*_*a*_ (M^−1^)	Δ*H* (kcal/mol)	−*T*Δ*S* (kcal/mol)	Δ*G* (kcal/mol)
1.46	2.34 ± 0.06 × 10^4^	80.0 ± 0.9	−88.3 ± 0.8	−8.32 ± 0.56

The calculated positive enthalpy change indicates an endothermic reaction counterbalanced with a high value of positive entropy change, making the spontaneous binding of kanamycin with TolC an entropy driven process.

### CD Measurements

**FIGURE 4:**
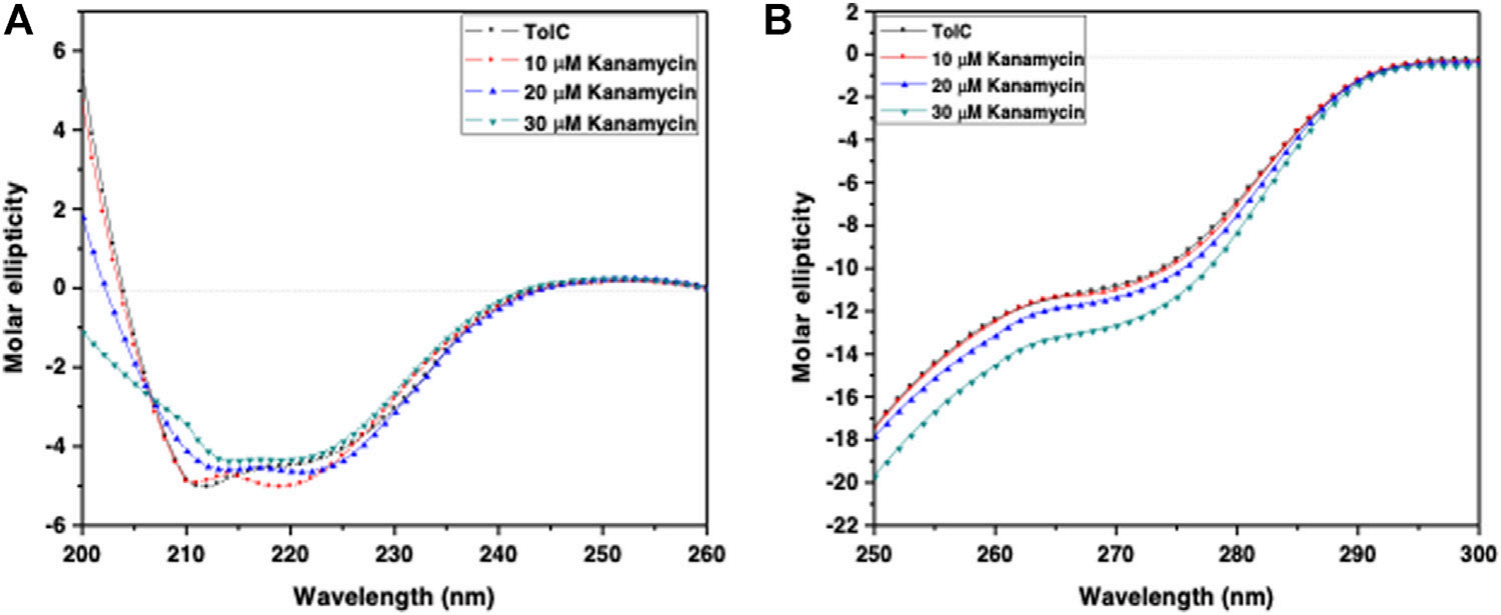
CD spectra of the purified outer membrane protein TolC without and with different concentrations of kanamycin measured in 20 mM phosphate buffer, added with 1% Octyl β-D-glucopyranoside, pH 7.2 at 25°C **(A)** Far UV-CD spectra of Tol C protein (■), and in the presence of increasing concentrations of kanamycin 10 µM (●), 20 µM (▲) and 30 µM (▼) **(B)** Near UV-CD spectra of TolC protein (■), and in the presence of increasing concentrations of kanamycin 10 µM (●), 20 µM (▲) and 30 µM (▼) respectively.

Circular dichroism (CD) is a relatively easy and highly applicable spectroscopic technique to study protein drug binding and the conformational changes (Rabbani et al., 2012; Varshney et al., 2010). It is also useful to understand the mode of interaction and determine the change in protein secondary structure (Rabbani and Choi, 2018; Rabbani et al., 2015). The intermolecular and intramolecular interactions involved in the protein secondary structure stability are affected upon binding with ligands or drug molecules (Varshney et al., 2014; Thakur et al., 2017). CD spectra is established with one positive spectra on 195 nm and two significant negative peaks centered on 209 nm and 222 nm in the far-UV region due to the n → π^*^ transition that shifts the peptide bond, which is characteristic of α-helix structure (Varlan and Hillebrand, 2010; Rogozea, 2012; Suryawashi, 2016; Zhang et at., 2016). To corroborate the fluorescence and ITC studies for kanamycin binding to TolC protein, CD measurements of TolC protein were carried out to observed the conformational behavior in the presence and absence of kanamycin. For all the experimental conditions, the protein concentration was kept constant at 10 μM. The change in ellipticity in the far-UV region from 200–240 nm provides information about the change in the regular secondary structure contents, such as α-helix and β-sheets of TolC, in the presence of kanamycin, which was used for deconvolution of regular secondary structure using the K2D2 online server (Perez-Iratxeta and Andrade-Navarro, 2008) and presented in Table 3. In Figure 4A, the binding of kanamycin with TolC indicates insignificant change in the secondary structure contents with a near decrease in the molar ellipticity in the range of 30 μM. However, the near-UV CD spectra in Figure 4B shows the change in the micro environment of the tryptophan residues around the binding site. The CD data indicates less flexible secondary structures with a more compact and stabilized complex in the presence of 30 μM kanamycin.

**TABLE 3:** Estimation of secondary structure content of TolC on addition of kanamycin.

Protein and antibiotics	α-Helix (%)	β-Sheets (%)
TolC	43.29	10.79
TolC +10 μM kanamycin	45.81	10.79
TolC +20 μM kanamycin	43.01	10.79
TolC +30 μM kanamycin	40.01	10.08

### Molecular Docking of Kanamycin with TolC

**FIGURE 5:**
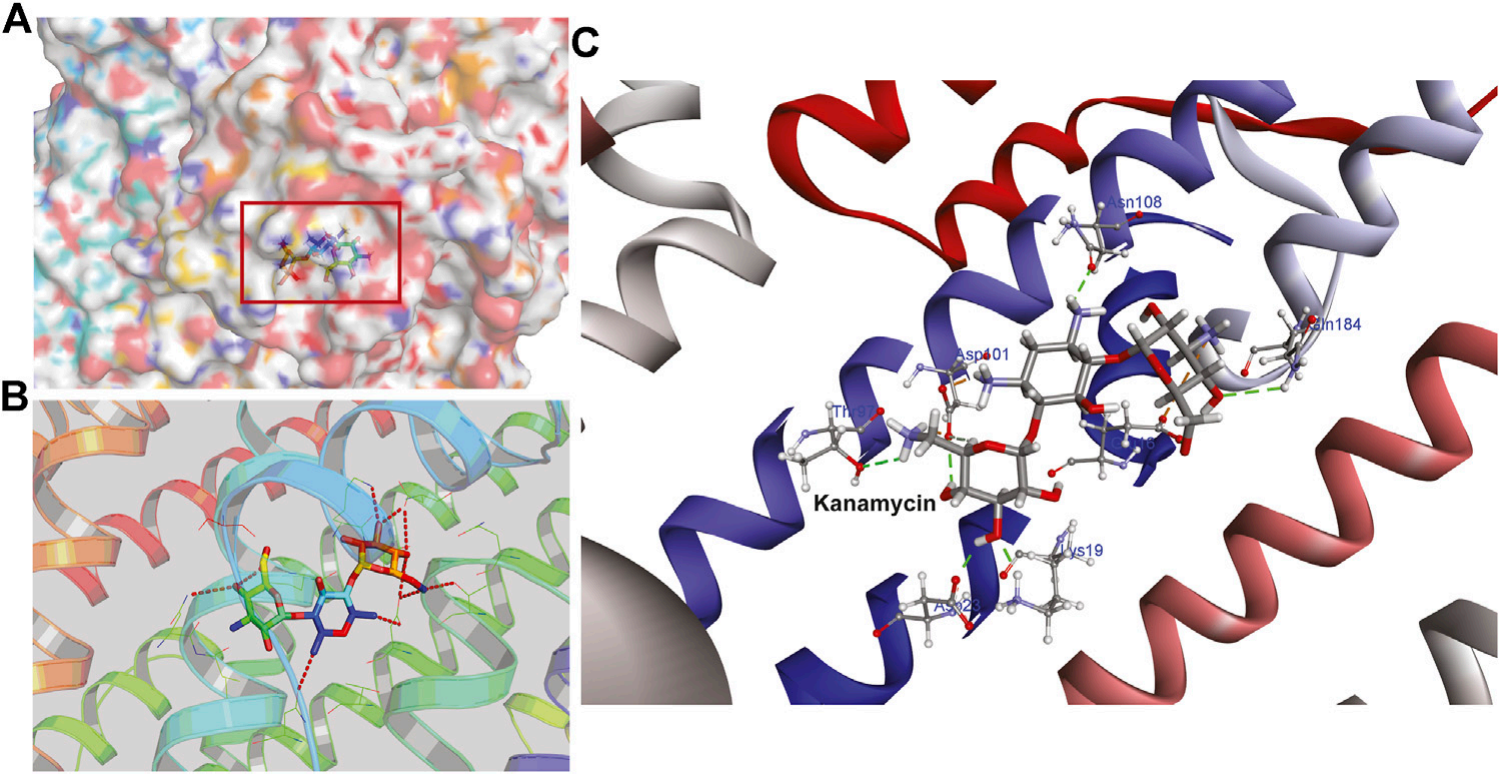
The structural representation of TolC residues interacting with kanamycin **(A)** surface potential view of TolC binding pocket (marked in square) occupied by kanamycin **(B)** ribbon diagram of TolC showing the interaction of docked kanamycin in the active site, and **(C)** amino acids residues with different positions (shown in gray sticks) involved in the binding with kanamycin (shown in gray and red sticks).

Hence, to analyze the interaction of kanamycin with TolC, we performed molecular docking (MD) to understand the nature and the mechanism of interaction at the molecular level. We identified that kanamycin interacts with TolC through a set of functionally active residues in the active site of the protein. The surface potential view in Figure 5A and the binding pocket view in Figure 5B illustrates the involvement of GLU16, LYS19, SER20, ASP23, THR97, ASP101, ASN108, and GLN189 amino acids in the active site of TolC, which interact with kanamycin; the residual atom involved and their hydrogen bonding lengths are presented in Table 4.

**TABLE 4:** The amino acid residues in the active site of TolC that are involved in the interaction with kanamycin.

Sl. No	Residue involved	Residues atom involved	H-bond length (Å)
1	GLU16	O	2.9
2	GLU16	O	2.7
3	LYS19	O	2.8
4	SER20	O	3.5
5	ASP23	O	2.7
6	ASP23	O	3.6
7	THR97	N	3.1
8	ASP101	O	3.2
9	ASP101	N	2.8
10	ASP101	N	2.8
11	ASN108	N	3.0
12	GLN184	O	3.5

**FIGURE 6:**
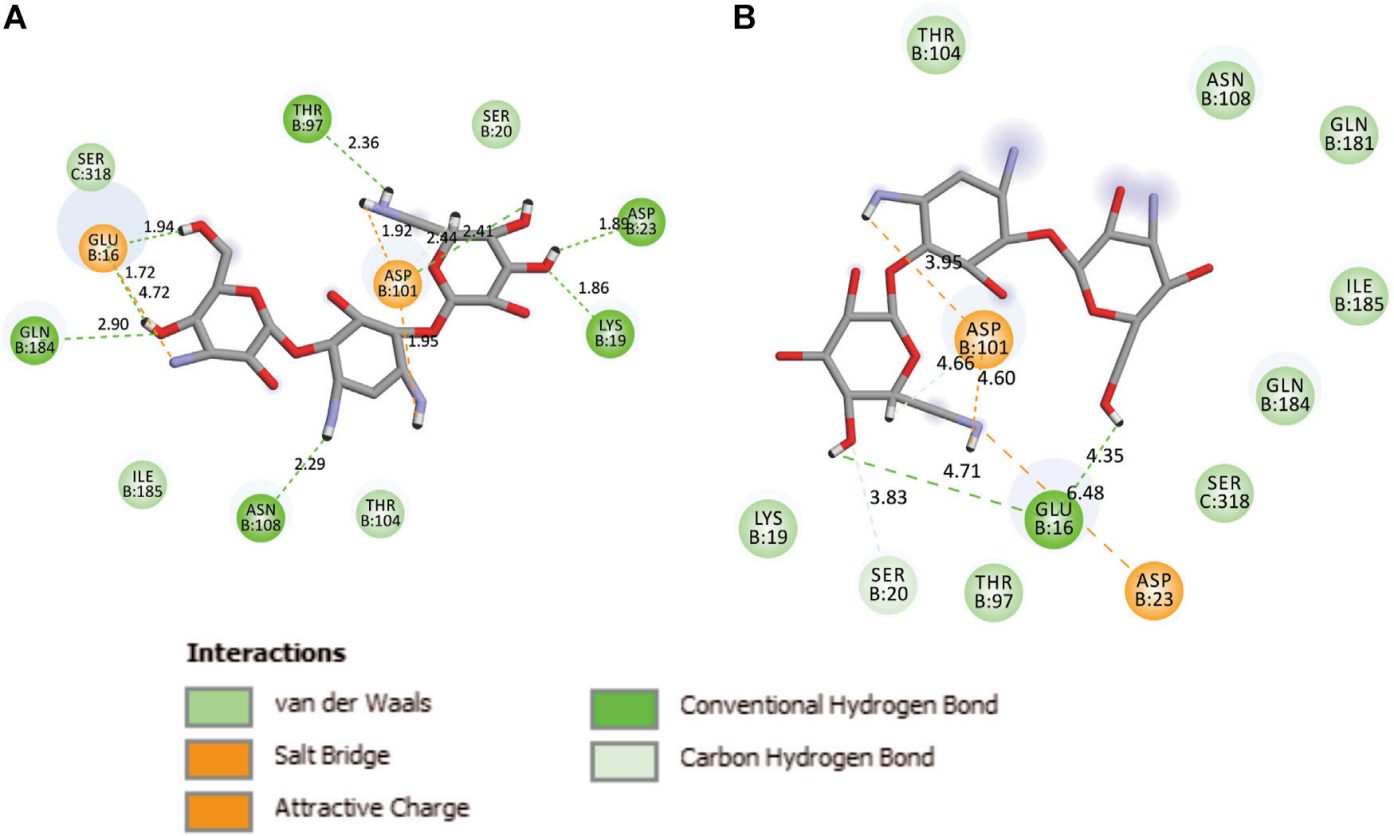
Two-dimensional representation of TolC-kanamycin interaction complex analysis. **(A)** TolC docked with kanamycin and stabilized through different interactions, and **(B)** the stabilizing interaction of residues through H-bonding (green color) and salt-bridging (orange color) in the active site of TolC protein.

During the MD, it was observed that the complex was stabilized by 12-hydrogen bonds, which involve five amino acid residues in the interactions. The hydrogen bonds are strong enough to keep hold tightly of kanamycin inside the active pocket of the protein. Similar salt bridge interactions were retained in the post docked structures and demonstrated that protein and kanamycin have high affinity and interact strongly. In addition, the system was also stabilized by non-polar interactions, such as pi-pi interactions, which were formed by residues with kanamycin [Figures 6A,B]. Interestingly, as compared to the docked conformation, the top ranked cluster representative complex showed loss of hydrogen bonding in the simulated complex which is due the structural reorientation of ligand and few residues of the protein in the binding site, thereby affecting the binding process.

### System Preparation

**FIGURE 7:**
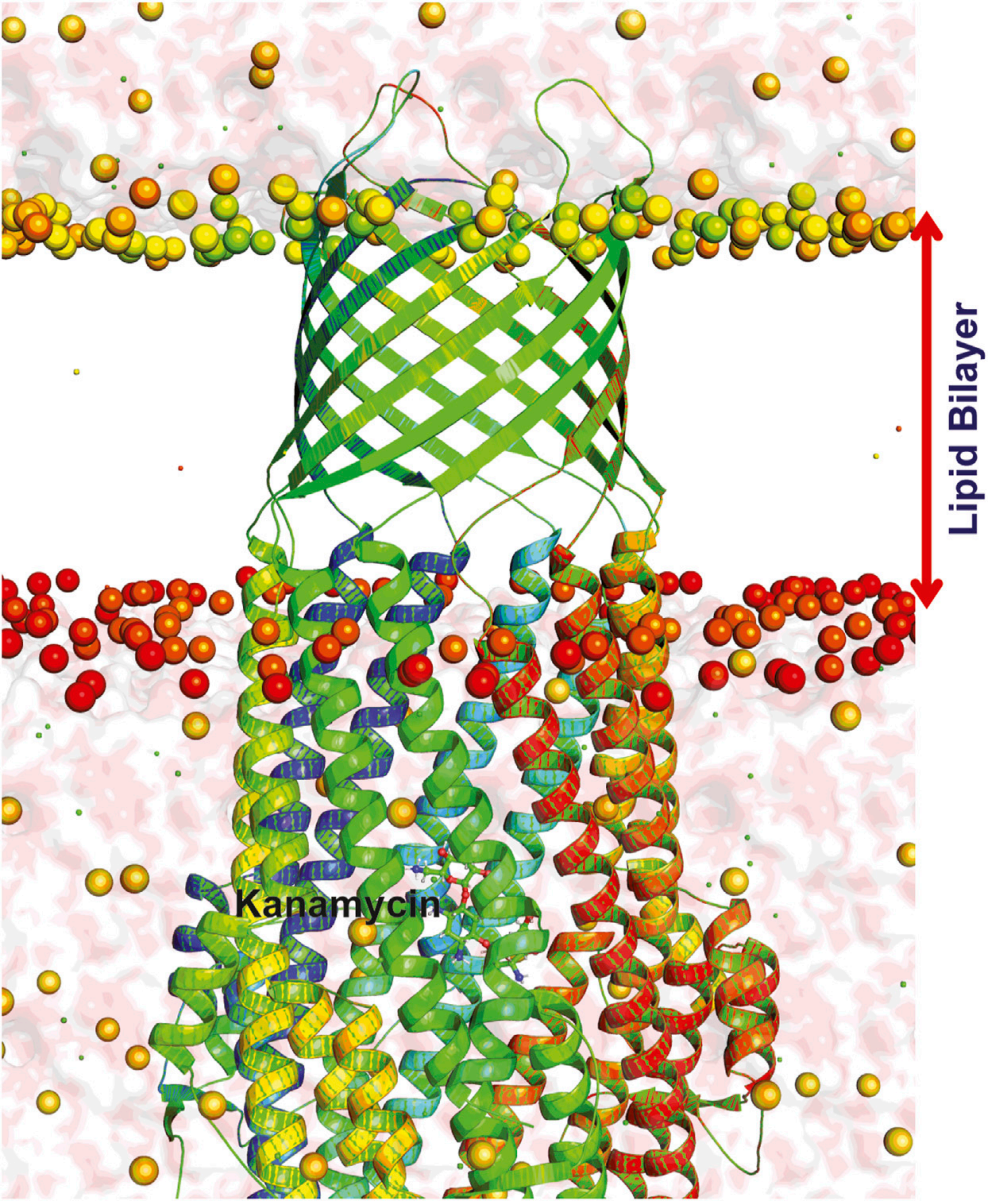
Dynamics of TolC-kanamycin complex in POPC lipid bilayer. The protomers are colored as blue, red, and green with the lipid bilayer of the bacterial outer membrane. The outer membrane embedded ß-barrel is open to the extracellular medium whereas the coiled coils taper close the periplasmic entrance of the α-helical barrel.

The best ranked pose i.e., 1EK9-kanamycin complex structure obtained from the docking simulations was subjected to 50 ns MD in lipid bilayer (Figure 7). The orientation of the 1EK9 structure with respect to the membrane was determined using the Positioning of Proteins in Membrane [PPM] server. The membrane-oriented protein-ligand was then inserted in the 1-palmitoyl-2-oleoyl-sn-glycero-3-phosphocholine [POPC] lipid bilayer using the CHARMM-GUI membrane builder. The protein-membrane system was solvated with TIP3P water model and 0.15 M NaCl and the ligand force field parameters were obtained using ParamChem with CHARMM General Force Field [CGenFF].

### Structural Changes in TolC on Kanamycin Binding

**FIGURE 8:**
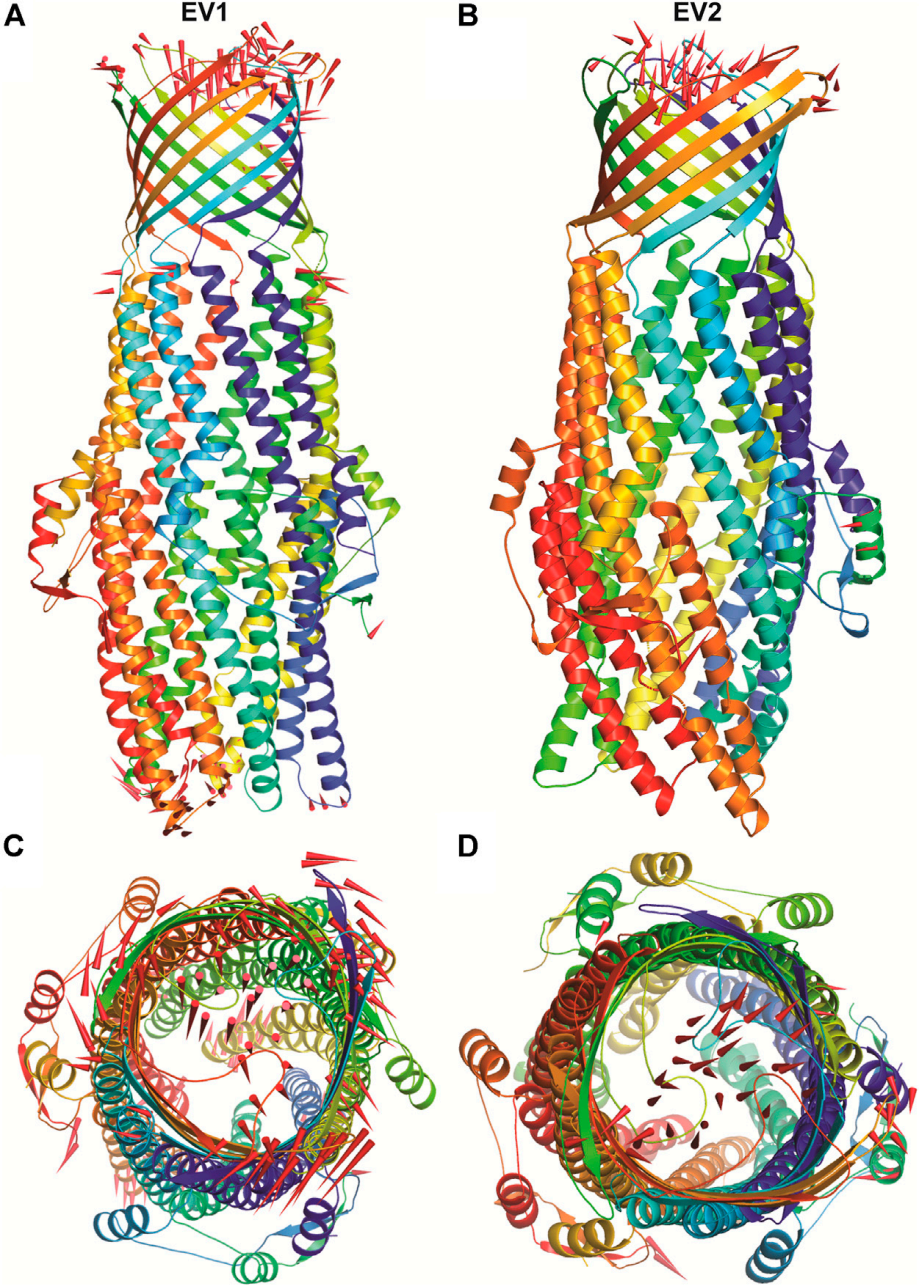
The overall architecture of TolC protein generated with RIBBONS (*p*). **(A)** and **(B)** are the *C*_*α*_ trace of TolC in closed and open form with individual promoters colored differently. The molecular threefold axis is aligned vertically, normal to the plane of the outer membrane. The β-barrel is at the top (distal) end and the *a*-helical domain is at the bottom (proximal) end. **(C)** and **(D)** are the exterior views of the proximal end of the α-helical barrel showing the coiled coils closing the proximal end of the tunnel and open state channel respectively. As PCA only describe the motion of main chain atoms of the complex the ligand (kanamycin) these structures could not be seen.

The structural motion and internal fluctuation of kanamycin bound form of TolC was analyzed through MD trajectory by essential dynamics. Initially, a diagonal covariance matrix from the main-chain of all atoms of the protein that captures the strenuous motion of the atom through eigenvectors and eigen values were inferred. The eigen values present the atomic contribution of motion (dynamics behavior and degree of fluctuations) while eigenvectors describe the overall direction of motion of the atoms. The first two eigenvectors (EV1 and EV2) obtained from ED analysis capture more than ∼74% of the total motion, indicating that these vectors define the essential subspace of the system [Figures 8A,B]. The trace value for the complex was computed to be 52.11 nm^2^. To quantitatively understand the movement directions captured by the eigenvectors, a porcupine plot was generated using the extreme projections on principal component PC1 and PC2. The direction of the arrow represents the direction of motion, while the length of the arrow characterizes the movement strength. The obtained plot suggests that rotational concerted movements are observed in two EVs, where the periplasmic and the extracellular ends of the protein display a high degree of inward movement, i.e. toward the pore.

To understand the structural diversity of the ensembles during MD, GROMOS method was employed to perform the clustering with a 0.2 nm cut off. The RMSD-based clustering generated a total of three clusters where the top ranked cluster harbors 2,293 structures (91.72% with an average RMSD of 0.175 nm), the second one occupies 199 structures (∼8% with RMSD of 0.148 nm), and the third has the least 9 structures with an average RMSD value of 0.157 nm.

### Dynamics of TolC-Kanamycin Complex

The binding of small ligands or drugs to a protein channel is a microscopic event that happens within fractions of a second. So, understanding molecular interactions and energetics of this binding is difficult to accomplish in detail. However, such complex processes can be investigated through molecular dynamics (MD) simulation studies (Dehury et al., 2017; Dehury et al., 2019), and the calculated root-mean-square deviation (rmsd) values can be used to estimate the structural dynamics of proteins at the atomic level (Gupta et al., 2017; Beg et al., 2018; Gulzar et al., 2019). MD simulation was performed with the top ranked conformation of kanamycin complex obtained from the molecular docking in POPC homogeneous lipid bilayer for 50 ns to assess all the structural dynamics through RMSDs calculations of the system. The system was comprised of 1,62,774 atoms with 238 POPC lipids, 36969TIP3 water molecules, 115 sodium, and 101 chlorine molecules at 303.15 K. The backbone RMSDs relative to the initial structure computed for the system have been depicted in Figure 5.

**FIGURE 9:**
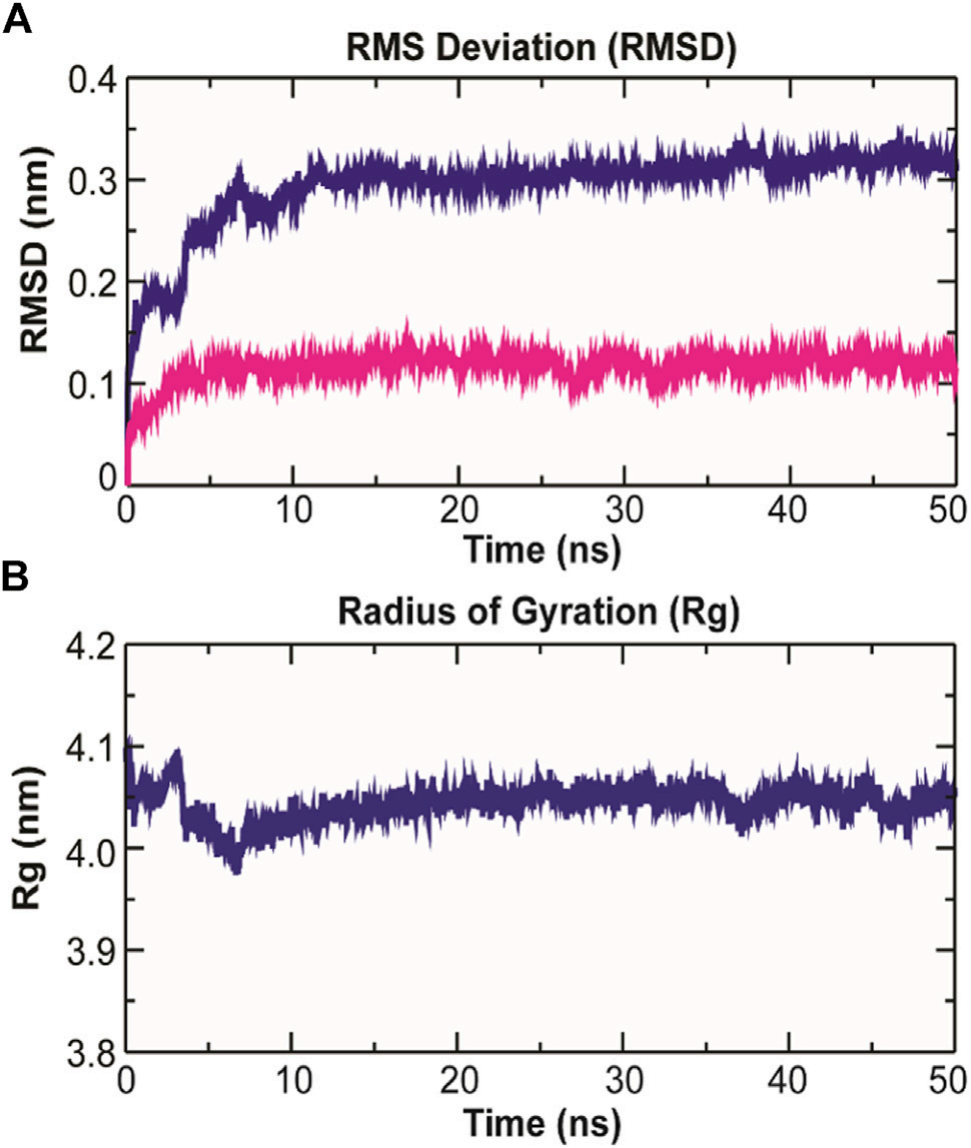
Structural dynamics of TolC on kanamycin binding. **(A)** The RMSD of the C_α_ atoms from their initial coordinator as function of time (ns). The blue line shows the RMSD for all the C_α_ atoms and the magenta line indicates the RMSD of the extracellular loops. **(B)** The radius of gyration (*R*_g_) of the C_α_ atoms from their initial coordinator as function of time (ns).

As evidenced from Figure 9A, the RMSD of ligand bound conformation of TolC shown in blue is mostly distributed within the range of 0.287 to 0.312 nm (average of ∼0.3 nm) with a constant trend indicating that the system reached equilibrium after 20 ns and maintained a stable trend till 50 ns. Like the protein-ligand complex, the RMSD of the ligand (magenta) also displayed a stable trend, which signifies that ligand within binding pocket remains stable throughout the MD. The radius of gyration (*R*_g_), which portrays the structure compactness and overall dimension of protein, is shown in Figure 9B. It represents the mass weighted root mean square distance of a collection of atoms from their common center of mass (Pathak et al., 2018). Here, the average value of *R*_g_ ∼ 4.06 nm achieved a stable trend after 20 ns, which indicates that the binding of kanamycin with TolC increases the compactness of the secondary structures packed into the 3D structure of protein.

**FIGURE 10:**
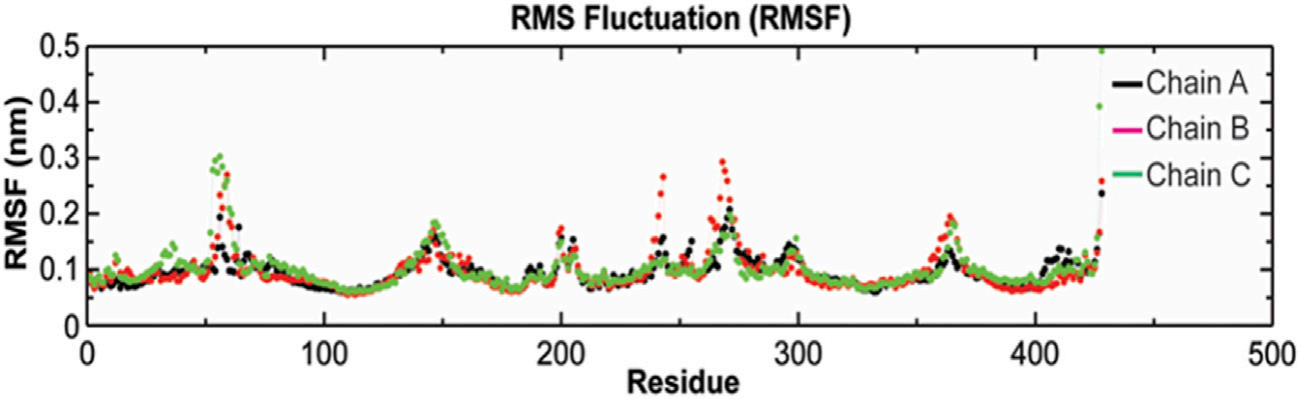
Root-mean-square fluctuations (RMSF) with respect to the residual dynamics of TolC protein on complexation with kanamycin.

The root-mean-square fluctuation (RMSF) of *C*_α_ atoms provides insights into the residual fluctuation and flexibility of the complex. The RMSF values of *C*_α_ atoms of each residue of three chains of the protein have been shown in Figure 10. The most flexible regions were located at the periplasmic and the extracellular ends of the protein with higher RMSF values. Except for these regions, including loops, most of the residues of the complex displayed a constant trend in RMSF. The ligand binding regions also displayed higher RMSF values with large peaks which reflect the flexibilities of binding site residue and their constant participation in the ligand recognition. The list of amino acids participating in ligand binding and their respective *C*_α_-RMSF values are represented in Table 5.

**TABLE 5:** RMSF values of *C*_α_ atoms of TolC residues binding with kanamycin.

Residues	RMSF (nm)
Asp101	0.0772
Glu16	0.0941
Lys19	0.0867
Gln184	0.0676
Thr97	0.08
Asn108	0.0624
Asp23	0.0818
Ser20	0.0819

### Intermolecular H-Bonding Analysis

**FIGURE 11:**
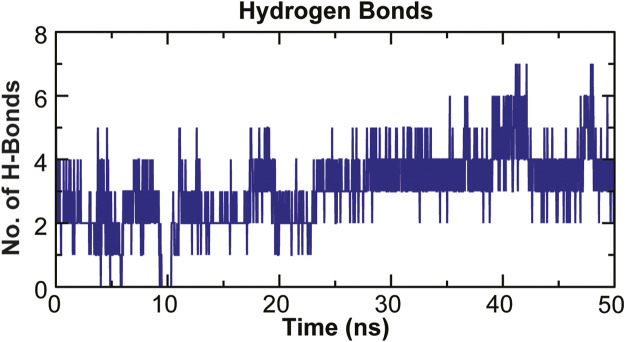
Stability of the TolC-kanamycin complex formed through intermolecular hydrogen bonding and their probable distributions in and around. The *y*-axis represents the number of hydrogen bonds forming with time (ns).

The stability of a biomolecular complex depends upon the intermolecular force of attraction, such as hydrogen bonding. The H-bonding formed in the protein-ligand complex and its stability can be explored to understand the molecular recognition and specificity of the interacting partners (Hubbard and Kamran Haider, 2001). Thus, the intermolecular hydrogen-bonding pattern in TolC protein and kanamycin complex was calculated using the *gmxh bond* tool by measuring the donor-acceptor distances during the MD simulations. Over the 50 ns time scale, TolC and kanamycin complex system exhibited differential intermolecular H-bond pattern with an average 2.86 hydrogen bonds per frame. The observed significant reduction in H-bonds in the complex after MD as compared to docked conformation were compensated by new H-bonds and electrostatic contacts, shown in Figure 11. Slight reorientation of the ligand within the binding pocket of TolC might be indicative of the foremost structural changes which might induce a significant decrease in the occupancy of the most H-bonds.

### Principal Component Analysis and Free Energy Landscape

**FIGURE 12:**
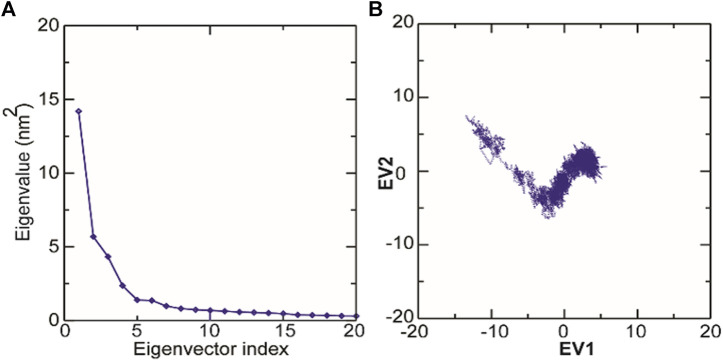
Molecular dynamics analysis of conformers of TolC. **(A)** The plot of eigenvalues corresponding to eigenvector index for the kanamycin complexed with TolC. **(B)** 2D projection of TolC along the EV1 and EV2 template for first two principal components.

Principal component analysis (PCA) is a robust statistical method that eases the convolution of a data set to extract biologically relevant movements of protein from irrelevant localized motions of atoms. Here, we visualized the sampled conformations in the subspace along the first two PCs using gmx anaeig in a two-dimensional projection, and porcupine plots were plotted (Figure 12) to visualize the motions. To illuminate the possible different conformations of the complex adopted during a simulation, free energy landscape (FEL) analysis was conducted using gmx sham module of GROMACS along the first two PCs. Here, we observed that TolC and kanamycin complex was flexible at both EVs and is also quite stable during the simulation after binding to kanamycin.

## Discussion

Antimicrobial export through the membrane bound efflux pumps is a frequent event that causes microbial resistance (Puzhao et al., 2016). In *E. coli,* the tripartite AcrAB-TolC efflux pump exports antibiotics and is the major cause of developing cross resistance (Nikaido and Zgurskaya., 2001). Studies on *E. coli* AcrAB-TolC efflux-protein complexed system have revealed that the apo-TolC mostly remain in the closed conformation, whereas the holo-TolC switches to interact with other partners, as seen in both the crystal and cryo-EM structures (Koronakis et al., 2000; Zhao et al., 2017). Hence, to get a detailed insight of the kanamycin binding with the outer membrane efflux protein TolC at a molecular level, we performed docking and molecular dynamic (MD) simulation studies. Analysis of the initial drug screened docking results revealed that kanamycin has a competitive docking score with a strong binding affinity with free energy of −6.5 kcal/mol towards TolC protein than other antibiotics.

The molecular weight of TolC obtained from the mascot MALDI mass spectroscopy results was found to be ∼54 kDa, which is the same as that earlier reported (Koronakis et al., 2000). The high affinity binding of kanamycin with TolC causes fluorescence intensities quenching. ITC data strengthens the TolC-kanamycin binding process through a single side binding endotherm, and the thermodynamics data obtained reveals an entropy driven stabilized binding process.

The intermolecular and intramolecular forces that stabilize the protein secondary and tertiary structures get affected when exposed to co-solutes or co-solvents (Kelly et al., 2005). The far-UV CD spectra of TolC on kanamycin binding showing the insignificant change in the α−helix and β−sheets contents of TolC protein indicates a high stable secondary structure. Whereas the near-UV CD spectra shows some changes in the tertiary structure that can be correlated with the structural fluctuation resulting from kanamycin binding and that facilitate TolC to interact with other binding partners in the triparty efflux pump complex.

The docking results have validated the molecular interaction and binding of kanamycin in the active pocket of TolC through the hydrogen bonding, van der Waal forces, and other expected interactions to form a stable TolC-kanamycin complex. The protein TolC was quite stable in the POPC lipid bilayer, and the conformational conversion in the MD study confirms the closed and open conformation as apo- and kanamycin-TolC complexes (Figures 8A,B), respectively. This conformational conversion has been reported to be mediated through an iris-like expansion of the periplasmic end, which is in fact necessary to maintain TolC-AcrA contact to permit the drug molecules to pass through the pump (Koronakis et al., 1997).

Further, we observed that the kanamycin binding with TolC is promoted through the inter-promoter hydrogen-bonding network involving five amino acid residues that are bound through twelve H-bonds and are mostly from the α-helices in the protein. The MD simulation data indicates the stability of these conformational changes occurring due to the kanamycin binding with TolC, which was further assessed by 50 nm MD simulation studies. The RMSD, *R*_g,_ and RMSF average values all together indicate that the binding of kanamycin with TolC stabilizes the structural complex with insignificant fluctuations. However, within 10 ns of initial simulations, some random fluctuation was observed that was stabilized up to 50 ns Thus, the kanamycin-TolC complex is quite stable after 10 ns of equilibration. The data altogether indicate that the entropy driven kanamycin binding to TolC results in the conformation fluctuation in the protein that favors the efflux. However, there are still limited experimental data to support the antibiotics binding to TolC and efflux across the *E. coli* membrane.

## Conclusion

We studied the binding of kanamycin with the outer membrane protein TolC and correlated its role in efflux. The results obtained using fluorescence, ITC and bioinformatics techniques indicated the preferential binding of kanamycin with TolC in the active-site and brings conformational changes in the protein. The far-UV CD results also supported the binding conformational changes in both the α-helix and β-sheets, which are part of the tunnel domain and the channel domain, respectively. This change in TolC conformation possibly regulates its interaction with other efflux pump partners (Koronakis et al., 2004). The binding of kanamycin with TolC and its efflux through the AcrAB-TolC system defines the cause of kanamycin resistance *E. coli* strains. Finally, the data altogether provides necessary information to establish the binding of kanamycin with TolC and thereby achieve efflux through the AcrAB-TolC channel. Thus, knowing the antibiotic binding and its efflux will help to discover potential antibacterial agents for the treatment of drug-resistant bacterial infections. However, this study needs further detailed analysis through different techniques to understand the mechanism of drug efflux in a more complete way.

## Data Availability

The raw data supporting the conclusions of this article will be made available by the authors, without undue reservation.

## References

[B1] Abdullah S. M. S. Fatma S. Rabbani G. Ashraf J. M. (2017). A spectroscopic and molecular docking approach on the binding of tinzaparin sodium with human serum albumin. J. Mol. Struct. 1127, 283–288. 10.1016/j.molstruc.2016.07.108

[B61] Aggen J. B. Armstrong E. S. Goldblum A. A. Dozzo P. Linsell M. S. Gliedt M. J. (2010). Synthesis and spectrum of the neoglycoside ACHN-490. Antimicrob. Agents Chemother. 54 (11), 4636–4642. 10.1128/AAC.00572-10 20805391 PMC2976124

[B2] Ahmad E. Rabbani G. Zaidi N. Ahmad B. Khan R. H. (2012). Pollutant-induced modulation in conformation and β-lactamase activity of human serum albumin. PLoS one 7 (6), e38372. 10.1371/journal.pone.0038372 22685563 PMC3369883

[B3] Ahmad E. Rabbani G. Zaidi N. Singh S. Rehan M. Khan M. M. (2011). Stereo-selectivity of human serum albumin to enantiomeric and isoelectronic pollutants dissected by spectroscopy, calorimetry and bioinformatics. PLoS one 6 (11), e26186. 10.1371/journal.pone.0026186 22073150 PMC3206814

[B4] Aono R. Tsukagoshi N. Yamamoto M. (1998). Involvement of outer membrane protein TolC, a possible member of the mar-sox regulon, in maintenance and improvement of organic solvent tolerance of *Escherichia coli* K-12. J. Bacteriol. 180 (4), 938–944. 10.1128/jb.180.4.938-944.1998 9473050 PMC106975

[B5] Baker C. J. M. D. Barrett F. F. M. D. Clark D. J. (1974). Incidence of kanamycin resistance among *Escherichia coli* isolates from neonates. J. Pediatr. 84, 126–130. 10.1016/s0022-3476(74)80573-1 12119935

[B62] Beg M. A. Thakur C. Meena L. S. (2018). Structural prediction and mutational analysis of Rv3906c gene of Mycobacterium tuberculosis H37Rv to determine its essentiality in survival. Adv. Bioinform. 2018, 1–12. 10.1155/2018/6152014 PMC6114228 30186322

[B6] Cagnacci S. Gualco L. Debbia E. Schito G. C. Marchese A. (2008). European emergence of ciprofloxacin-resistant *Escherichia coli* clonal groups O25:H4-ST 131 and O15:K52:H1 causing community-acquired uncomplicated cystitis. J. Clin. Microbiol. 46 (8), 2605–2612. 10.1128/JCM.00640-08 18579721 PMC2519467

[B7] Dehury B. Behera S. K. Mahapatra N. (2017). Structural dynamics of Casein Kinase I (CKI) from malarial parasite Plasmodium falciparum (Isolate 3D7): insights from theoretical modelling and molecular simulationsPlasmodium falciparum (Isolate 3D7): insights from theoretical modelling and molecular simulations. J. Mol. Graphics Model. 71, 154–166. 10.1016/j.jmgm.2016.11.012 27923179

[B8] Dehury B. Tang N. Kepp K. P. (2019). Molecular dynamics of C99-bound γ-secretase reveal two binding modes with distinct compactness, stability, and active-site retention: implications for Aβ production. Biochem. J. 476, 1173–1189. 10.1042/bcj20190023 30910800

[B63] Delmar J. A. Su C.-C. YU E. W. (2014). Bacterial multidrug efflux transporters. Ann. Rev. Biophys. 43, 93–117. 10.1146/annurev-biophys-051013-022855 24702006 PMC4769028

[B9] Dinesh K. B. Vignesh K. P. Bhubaneswar S. P. Mitra A. (2010). Drugs rules and regulations: different countries (India, China, Russia and United States) - a review. Int. J. Pharm. Pharma Sci. 2, 16.

[B10] Dinh T. Paulsen I. T. Saier M. H. Jr (1994). A family of extracytoplasmic proteins that allow transport of large molecules across the outer membranes of gram-negative bacteria. J. Bacteriol. 176, 3825–3831. 10.1128/jb.176.13.3825-3831.1994 8021163 PMC205578

[B11] Du D. van Veen H. W. Luisi B. F. (2015). Assembly and operation of bacterial tripartite multidrug efflux pumps. Trends Microbiol. 23, 311–319. 10.1016/j.tim.2015.01.010 25728476

[B12] Fralick J. A. (1996). Evidence that TolC is required for functioning of the mar/AcrAB efflux pump of *Escherichia coli* . J. Bacteriol. 178, 5803–5805. 10.1128/jb.178.19.5803-5805.1996 8824631 PMC178425

[B13] Friesner R. A. Banks J. L. Murphy R. B. Halgren T. A. Klicic J. J. Mainz D. T. (2004). Glide: a new approach for rapid, accurate docking and scoring. 1. Method and assessment of docking accuracy. J. Med. Chem. 47, 1739–1749. 10.1021/jm0306430 15027865

[B66] Gulzar A. Borau L. V. Buchenberg S. Wolf S. Stock D. (2019). Energy transport pathways in proteins: a non-equilibrium molecular dynamics simulation study. J. Chem. Theory Comput. 15 (10), 5750–5757. 10.1021/acs.jctc.9b00598 31433644

[B67] Gupta A. Mishra S. Singh S. Mishra S. (2019). (2017). Prevention of IcaA regulated poly N-acetyl glucosamine formation in Staphylococcus aureus biofilm through new-drug like inhibitors: in silico approach and MD simulation study. Microb. Pathog. 110, 659–669. 10.1016/j.micpath.2017.05.025 28579399

[B14] Hubbard R. E. Kamran Haider M. (2001). Hydrogen Bonds in Proteins: role and Strength. eLS. John Wiley & Sons. 10.1002/9780470015902.a0003011.pub2

[B15] Johnson W. C. Jr. (1990). Protein secondary structure and circular dichroism: a practical guide. Proteins 7, 205–214. 10.1002/prot.340070302 2194218

[B16] Kelly S. M. Jess T. J. Price N. C. (2005). How to study proteins by circular dichroism. Biochim. Biophys. Acta (Bba) - Proteins Proteomics 1751, 119–139. 10.1016/j.bbapap.2005.06.005 16027053

[B17] Koronakis V. Eswaran J. Hughes C. (2004). Structure and function OF tolc: the bacterial exit duct for proteins and drugs. Annu. Rev. Biochem. 73, 467–489. 10.1146/annurev.biochem.73.011303.074104 15189150

[B18] Koronakis V. Li J. Koronakis E. Stauffer K. (1997). Structure of TolC, the outer membrane component of the bacterial type I efflux system, derived from two-dimensional crystals. Mol. Microbiol. 23 (3), 617–626. 10.1046/j.1365-2958.1997.d01-1880.x 9044294

[B19] Koronakis V. Sharff A. Koronakis E. Luisi B. Hughes C. (2000). Crystal structure of the bacterial membrane protein TolC central to multidrug efflux and protein export. Nature 405, 914–919. 10.1038/35016007 10879525

[B20] Lakowicz Joseph. R. (2006). Principle of fluorescence spectroscopy. Boston, MA: Springer, 277–330.

[B69] Landman D. Babu E. Shah N. Kelly P. Bäcker M. Bratu S. (2010). Activity of a novel aminoglycoside, ACHN-490, against clinical isolates of Escherichia coli and Klebsiella pneumoniae from New York City. J. Antimicrob. Chemother. 65 (10), 2123–2127. 10.1093/jac/dkq278 20667885

[B21] Li X. Z. Nikaido H. (2009). Efflux-mediated drug resistance in bacteria: an update. J. Bacteriol. 69, 1555–1623. 10.2165/11317030-000000000-00000 PMC2847397 19678712

[B70] Michael C. A. Dominey-Howes D. Labbate M. (2014). The antimicrobial resistance crisis: causes, consequences, and management. Front. Pub. Health 2 (145), 1–8. 10.3389/fpubh.2014.00145 25279369 PMC4165128

[B71] Money P. Kelly A. F. Gould W. J. Denholm-Price J. Threlfall E. J. Fielder M. D. (2010). Cattle, weather and water: mapping Escherichia coli O157:H7 infections in humans in England and Scotland. Env. Microbiol. 12 (10), 2633–2644. 10.1111/j.1462-2920.2010.02293.x 20642796

[B22] Morona R. Manning P. A. Reeves P. (1983). Identification and characterization of the ToIC protein, an outer membrane protein from *Escherichia coli* . J. Bacteriol. 153, 693–699. 10.1128/JB.150.3.1016-1023.1982 6337123 PMC221686

[B23] Morona R. Reeves P. (1981). Molecular cloning of the *tolC* locus of *Escherichia coli* K-12 with the use of transposon Tn*10* . Mol. Gen. Genet. 184, 430–433. 10.1007/BF00352517 6278256

[B24] Morona R. Reeves P. (1982). The *tolC* locus of *Escherichia coli* affects the expression of three major outer membrane proteins. J. Bacteriol. 150, 1016–1023. 10.1128/JB.150.3.1016-1023.1982 6281230 PMC216317

[B25] Nidaido H. (1994). Prevention of drug access to bacteria targets: permeability barriers and active efflux. Science 264, 382–388. 10.1126/science.8153625 8153625

[B26] Nikaido H. (1998). Multiple antibiotic resistance and efflux. Curr. Opin. Microbiol. 1, 516–523. 10.1016/s1369-5274(98)80083-0 10066525

[B27] Nikaido H. Zgurskaya H. I. (2001). AcrAB and related multidrug efflux pumps of *Escherichia coli* . J. Mol. Microbiol. Biotechnol. 3, 215–218. 10.1159/000103594 11321576

[B28] Nishino K. Yamada J. Hirakawa H. Hirata T. Yamaguchi A. (2003). Roles of TolC-dependent multidrug transporters of *Escherichia coli* in resistance to β-lactams. Antimicrobe Agent Chemother. 47, 3030–3033. 10.1128/AAC.47.9.3030-3033.2003 PMC182617 12937021

[B29] Pathak R. K. Gupta A. Shukla R. Baunthiyal M. (2018). Identification of new drug-like compounds from millets as Xanthine oxidoreductase inhibitors for treatment of Hyperuricemia: a molecular docking and simulation study. Comput. Biol. Chem. 76, 32–41. 10.1016/.j.compbiolchem.2018.05.015 29906649

[B30] Paulsen I. T. Brown M. H. Skurray R. A. (1996). Proton-dependent multidrug efflux systems. Microbiol. Rev. 60 (4), 575–608. 8987357 10.1128/mr.60.4.575-608.1996 PMC239457

[B31] Paulsen I. T. Park J. H. Choi P. S. Saier M. H. Jr. (1997). A family of Gram-negative bacterial outer membrane factors function in the export of proteins, carbohydrates, drugs and heavy metals from Gram-negative bacteria. FEMS Microbiol. Lett. 156, 1–8. 10.1111/j.1574-6968.1997.tb12697.x 9368353

[B64] Pennington T. H. (2014). *E. coli* O157 outbreaks in the United Kingdom: past, present, and future. Infect. Drug Resist. 7, 211–222. 10.2147/IDR.S49081 25187729 PMC4149388

[B32] Perez-Iratxeta C. Andrade-Navarro M. A. (2008). K2D2: estimation of protein secondary structure from circular dichroism spectra. BMC Struct. Biol. 8 (25), 1–5. 10.1186/1472-6807-8-25 18477405 PMC2397409

[B65] Poole K. (2007). Efflux pumps as antimicrobial resistance mechanisms. Ann. Med. 39 (3), 162–176. 10.1080/07853890701195262 17457715

[B33] PuZhao Y. Z. Li Y. Zou J. Ma Q. Zhao Y. Ke Y. (2016). Enhanced efflux activity facilitates drug tolerance in dormant bacterial cells. Mol. Cel 62, 737–775. 10.101/j.molcel.2016.03.035 PMC4850422 27105118

[B34] Rabbani G. Ahmad E. Khan M. V. Ashraf M. T. Bhat R. Khan R. H. (2015). Impact of structural stability of cold adapted *Candida antartica* lipase B (CaLB): in relation to pH, chemical and thermal denaturation. RSC Adv. 5 (26), 20115–20131. 10.1039/C4RA17093H

[B35] Rabbani G. Ahmad E. Zaid N. Khan R. H. (2011). pH-dependent conformational transitions in conalbumin (ovotransferrin), a metalloproteinase from hen egg white. Cell Biochem Biophys 61, 551–560. 10.1007/s12013-011-9237-x 21833676

[B36] Rabbani G. Ahmad E. Zaidi N. Fatima S. Khan R. H. (2012). pH-induced motel globule state of *Rhizopus niveus* lipase is more resistance against thermal and chemical denaturation than its native state. Cel Biochem Biophys 62, 487–499. 10.1007/s12013-011-9335-9 22215307

[B37] Rabbani G. Choi I. (2018). Roles of osmolytes in protein folding and aggregation in cells and their biotechnology applications. Int. J. Biol. Macromol 109, 483–491. 10.1016/j.ijbiomac.2017.12.100 29274422

[B38] Rabbani G. Kaur J. Ahmad E. Khan R. Jain S. K. (2013). Structural characteristics of the thermostable immunogenic outer membrane protein from *Salmonella enterica* serovar Typhi. Appl. Microbiol. Biotechnol. 98, 2533–2543. 10.1007/s00253-013-5123-3 23949993 PMC7080034

[B39] Rahman S. Rehman M. T. Rabbani G. Khan P. AlAjmi M. F. Hassan M. I. (2019). Int. J. Mol. Sci. 20, 2727. 10.3390/ijms20112727 PMC6600547 31163649

[B68] Ristuccia A. M. Cunha B. A. (1985). An overview of Amikacin. Therap. Drug Monitor. 7 (1), 12–25. 10.1097/00007691-198503000-00003 3887667

[B40] Rogozea A. (2012). EPR and circular dichroism solution studies on the interactions of bovine serum albumin with ionic surfactants and β-cyclodextrin. J. Phys. Chem. B 116, 14245–14253. 23163315 10.1021/jp308650r

[B41] Sulavik M. C. Houseweart C. Cramer C. Jiwani N. MurgoloGreen N. J. DiDomenico B. (2001). Antimicrobe Agents Chemother. 45, 1126–1136. 10.1128/AAC.45.4.1126-1136.2001 PMC90435 11257026

[B42] Suryawanshi V. D. Walekar L. S. Gore A. H. Anbhule P. V. Kolekar G. B. (2016). Spectroscopic analysis on the binding interaction of biologically active pyrimidine derivative with bovine serum albumin. J. Pharm. Anal. 6, 56–63. 10.1016/j.jpha.2015.07.001 29403963 PMC5762442

[B43] Thakur K. Kaur T. Singh J. Rabbani G. Khan R. H. Hora R. (2017). *Sauromatum guttatum* lectin: spectral studies, lectin-carbohydrate interaction, molecular cloning and *in silico* analysis. Int. J. Biol. Macromol 104, 1267–1279. 10.1016/j.ijbiomac.2017.06.123 28684356

[B44] Tikhonova E. B. Yamada Y. Zgurskaya H. I. (2011). Sequential mechanism of assembly of multidrug efflux pump. Acrab-tolc. *Chem. Biol.* 18, 454–463. 21513882 10.1016/j.chembiol.2011.02.011 PMC3082741

[B45] Varlan A. Hillebrand M. (2010). Bovine and human serum albumin interactions with 3-carboxyphenoxathiin studied by fluorescence and circular dichroism spectroscopy. Molecules 15, 3905–3919. 10.3390/molecules15063905 20657416 PMC6257641

[B46] Varshney A. Ahmad B. Rabbani G. Kumar V. Yadav S. Khan R. H. (2010). Acid-induced unfolding of didecameric keyhole limpet hemocyanin: detection and characterizations of decameric and tetrameric intermediate states. Amino Acids 39, 899–910. 10.1007/s00726-010-0524-4 20213446

[B47] Varshney A. Rabbani G. Badr Gamal. Khan R. H. (2014). Cosolvents induced unfolding and aggregation of keyhole limpet hemocyanin. Cel Biochem Biophys 69, 103–113. 10.1007/s1203-013-97764-4 24242285

[B48] Wang H. Dzink-Fox J. L. Chen M. Levy S. B. (2001). Genetic characterization of highly fluoroquinolone-resistant clinical *Escherichia coli* strains from China: role ofacrR mutations. Antimicrob. Agents Chemother. 45, 1515–1521. 10.1128/AAC.45.5.1515-1521.2001 11302820 PMC90498

[B49] Zgurskaya H. I. (2009). Multicomponent drug efflux complexes: architecture and mechanism of assembly. Future Microbiol. 4, 919–932. 10.2217/fmb.09.62 19722844 PMC2763196

[B50] Zgurskaya H. I. Nikaido H. (1999). Bypassing the periplasm: reconstitution of the AcrAB multidrug efflux pump of *Escherichia coli* . Proc. Natl. Accad. Sci. U.S.A. 96, 7190–7195. 10.1073/pnas.96.13.7190 PMC22048 10377390

[B51] Zhang D. Li H. Lin X. Peng X. (2015). Outer membrane proteomics of kanamycin-resistant *Escherichia coli* identified MipA as a novel antibiotic resistance-related protein. FEMS Microbiol. Lett. 362, 1–8. 10.1093/femsle/fnv074 25940639

[B52] Zhang L. Sahu I. D. Xu M. Wang Y. Hu X. (2016). Data for β-lactoglobulin conformational analysis after (-)-epigallocatechingallate and metal ions binding. Data Brief 10, 474–477. 10.1016/j.dib.2016.12.021 28054010 PMC5198795

[B53] Zhao W. Guizhen F. Corey F. H. James N. B. Irina I. S. Michael F. S. (2017). An allosteric transport mechanism for the AcrAB-TolC multidrug efflux pump. eLife 29 (6), e24905. 10.7554/eLife.24905 PMC5404916 28355133

