# Supplementary material for: Kanamycin-Mediated Conformational Dynamics of Escherichia coli Outer Membrane Protein TolC
Source: Front Mol Biosci. 2021 Apr 15;8:636286. doi: 10.3389/fmolb.2021.636286 (PMC8083960; doi:10.3389/fmolb.2021.636286)
Supplement: Supplementary file 1 [file datasheet1.pdf]

Kanamycin mediated conformational dynamics of *Escherichia coli* outer membrane protein TolC

Biraja S. Pattanayak[1], Budheswar Dehury[2], Mamali Priyadarshinee[3], Suman Jha[4], Tushar K. Beuria[5], Dhananjay Soren[1], Bairagi C. Mallick[6*]

[1]Department of Zoology, Ravenshaw University, Cuttack-753003, Odisha, India.
[2]Department of Biochemistry, University of Cambridge, Cambridge CB2 1GA, UK.
[3]Department of Life Sciences, Ravenshaw University, Cuttack-753003, Odisha, India.
[4]Department of Life Sciences, National Institute of Technology, Rourkela-769008, Odisha.
[5]Institute of Life Sciences, Nalco Square, Bhubaneswar-751023, Odisha.
[6]Department of Chemistry, Ravenshaw University, Cuttack-753003, Odisha.

*** Correspondence:**
Dr. Bairagi C. Mallick,
Department of Chemistry, Ravenshaw University, Cuttack-753003, Odisha, India
E-mail: bcmallick@ravenshawuniversity.ac.in

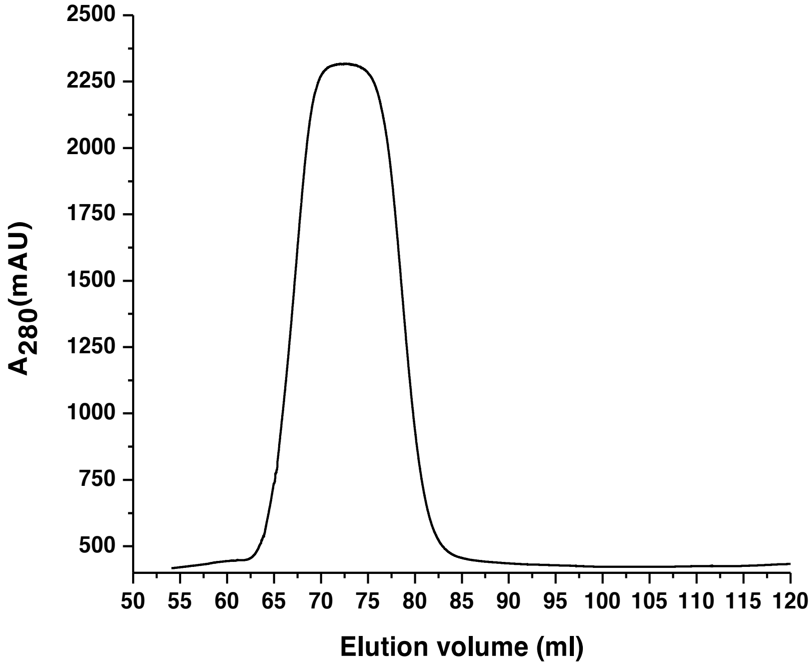

Figure SI-1: FPLC chromatogram of the purified recombinant TolC protein using the AKTA Explorer chromatography system (GE Healthcare, USA) at 4°C. A superdex-200 prepacked column was used to perform the protein purification and the desired fractions were collected for further use.

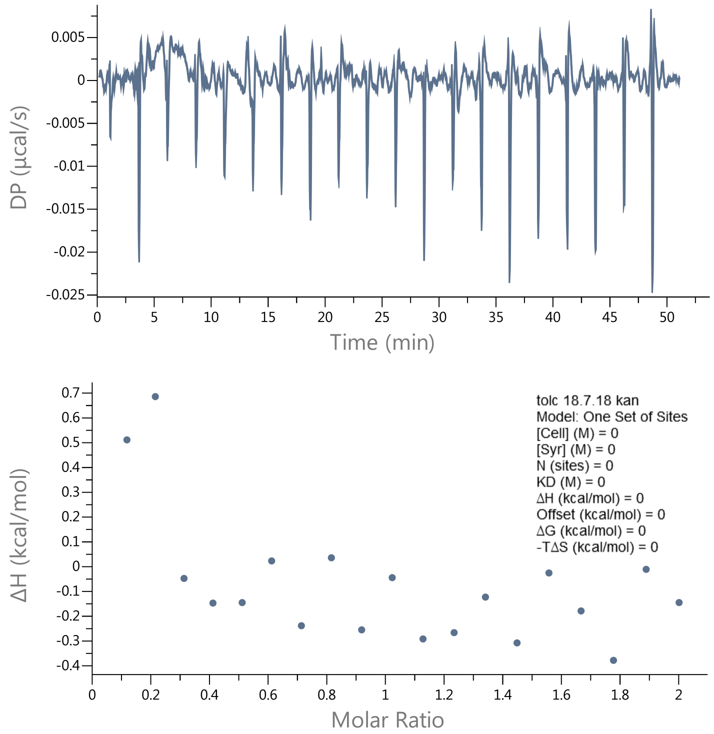

Figure SI-2: ITC thermogram of TolC at 298.15 K. The top panel shows the raw data of the injection profile and the bottom panel represents the calorimetric binding isotherm. The y-axis is the normalized heat of binding for each injection of ligands in x-axis. The inset represents the thermodynamic parameters.

Table SI-I: Table showing the MALDI mass spectroscopy data for the purified recombinant TolC protein.

Search title : ILS Proteomics2\ILS-117-09062016\MSMS 6\A7

Database : UniProtKB-SwissProt sprot_20150527 (548586 sequences; 195452300 residues)

Taxonomy : Escherichia coli (26554 sequences)

	Spot ID	Best Protein Accession	Best Protein Mass	Best Protein Score	Best Protein Description
b-gal	A2	BGAL_ECOLI	117321	1510	Beta-galactosidase OS=Escherichia coli (strain K12) GN=lacZ PE=1 SV=2
Tolc-1	A7	TOLC_ECOLI	53708	108	Outer membrane protein TolC OS=Escherichia coli (strain K12) GN=tolC PE=1 SV=3
	A8	TNR0_ECOLX	21521	37	Transposon Tn2501 resolvase OS=Escherichia coli GN=tnpR PE=3 SV=1
	A9	TOLC_ECOLI	53708	86	Outer membrane protein TolC OS=Escherichia coli (strain K12) GN=tolC PE=1 SV=3
Tolc-2	A10	TOLC_ECOLI	53708	158	Outer membrane protein TolC OS=Escherichia coli (strain K12) GN=tolC PE=1 SV=3
	A11	TOLC_ECOLI	53708	85	Outer membrane protein TolC OS=Escherichia coli (strain K12) GN=tolC PE=1 SV=3
	A12	TOLC_ECOLI	53708	117	Outer membrane protein TolC OS=Escherichia coli (strain K12) GN=tolC PE=1 SV=3

Search Parameters

Type of search : Sequence Query

Enzyme : Trypsin

Fixed modifications : Carbamidomethyl (C)

Variable modifications : Deamidated (NQ),Oxidation (M)

Mass values : Monoisotopic

Protein Mass :

Unrestricted

Peptide Mass Tolerance : ± 100 ppm

Fragment Mass Tolerance: ± 0.8 Da

Max Missed Cleavages : 1
